# Avoiding sexual interference: herkogamy and dichogamy in style dimorphic flowers of *Narcissus broussonetii* (Amaryllidaceae)

**DOI:** 10.1093/aobpla/plz038

**Published:** 2019-08-09

**Authors:** Daniel Barranco, Juan Arroyo, Rocío Santos-Gally

**Affiliations:** 1 Departamento de Biología Vegetal y Ecología, Universidad de Sevilla, apartado, Sevilla, Spain; 2 CONACyT-Instituto de Ecología, A.P. 70-275, Ciudad Universitaria, Universidad Nacional Autónoma de México, Ciudad de México, México

**Keywords:** dichogamy, floral morphology, flower development, herkogamy, heterostyly, heterozygosity, *Narcissus broussonetii*, self-interference, style dimorphism

## Abstract

Spatial (herkogamy) or temporal (dichogamy) separation of sex organs are mechanisms considered to restrict self-pollination and promote outcrossing. Additionally, avoidance of self-interference is proposed to be the driving force for the evolution of these mechanisms, particularly in self-incompatible species. However, species with anthers and stigmas at different levels may increase the rate of imprecise pollen transfer, resulting in pollen discounting. Non-reciprocal stylar dimorphism has been considered a transitional, unstable stage towards the evolution of reciprocal style dimorphism (distyly), to simultaneously avoid interference and lack of precision. In this study we investigate the spatial and temporal separation of sex organs in a population of the style dimorphic and self-incompatible *Narcissus broussonetii* and their consequences in the reciprocity between the sex organs of morphs and their fecundity. First, we evaluated the relative growth of sex organs after anthesis. Then, we studied the stigma receptivity along the flower lifespan including its effect on seed production in both morphs. Finally, given the weak reciprocity between the sex organs of morphs of this species, we estimated population genetic diversity parameters in Long- and Short-styled plants to explore differences between them as a result of rates of inbreeding due to different mating strategies. We observed that Long-styled plants and Short-styled plants present different strategies to avoid sexual interference and both of them had negative consequences in the reciprocity between the sex organs of morphs. Long-styled plants exhibited a delay in stigma receptivity and a higher growth rate of the style after anthesis, while Short-styled plants presented higher herkogamy and no delay in stigma receptivity. These findings suggest that the avoidance of self-interference, in stylar dimorphic *Narcissus* species, seems to be more critical than improving of reciprocity between the sex organs of morphs. This might explain why reciprocal herkogamy (distyly) is rare in the genus.

## Teaser

In this study we investigated the spatial and temporal separation of sex organs in a population of the style dimorphic and self-incompatible *Narcissus broussonetii* and their consequences in the reciprocity between the sex organs of morphs and their fecundity. We observed that Long-styled plants and Short-styled plants present different strategies to avoid sexual interference and both of them had negative consequences in the reciprocity between the sex organs of morphs. Long-styled plants exhibited a delay in stigma receptivity and a higher growth rate of the style after anthesis, while Short-styled plants presented higher herkogamy and no delay in stigma receptivity. These findings suggest that the avoidance of self-interference in stylar dimorphic *Narcissus* species seems to be more critical than improving reciprocity between the sex organs of morphs.

## Introduction

Flowering plants exhibit an extraordinary diversity of flower forms, many of which are related to variations in the morphology and arrangement of sex organs (Darwin 1877; Barrett 2002). The most predominant sexual condition in angiosperms is hermaphroditism, the presence of both sex organs (pistil and stamens) within the same flower. Hermaphroditic flowers present some advantages, such as resource economy for the plant because traits to attract pollinators serve the dual role of promoting female and male floral functions (Givnish 1980; Charlesworth and Charlesworth 1987b; Lloyd 1987). However, the proximity of the sex organs may increase the risk of self-pollination with the possible detrimental consequences of pollen discounting (Harder and Wilson 1998) and inbreeding depression (Darwin 1876; Charlesworth and Charlesworth 1987a). To prevent self-pollination, plants have evolved floral forms where sexual organs are separated spatially (herkogamy) or temporarily (dichogamy) within the hermaphrodite flower (Lloyd and Webb 1986; Webb and Lloyd 1986). Both mechanisms are widespread among angiosperms and often have been considered to restrict self-fertilization and promote outcrossing (Darwin 1876; Charlesworth and Charlesworth 1987a; Barrett 2002).

Nevertheless, dichogamy and herkogamy also occur in plant species having physiological self-incompatibility (Lloyd and Webb 1986; Webb and Lloyd 1986), suggesting that inbreeding depression is not necessarily an explanation for the maintenance of these traits, and therefore factors related to male and female functions in pollination become more relevant. Thus, an alternative hypothesis is that temporal and spatial sex separation may decrease self-interference (i.e. the conflict between the removal and deposition of pollen; Webb and Lloyd 1986). Because reproduction is energetically expensive, self-interference may potentially damage the female and male reproductive success. Sexual interference may be related with physiological or merely physical interactions between the sexual organs. Physiological interactions affect the female and male function and may involve ovule discounting in species with ovarian self-incompatibility, as self-pollen tubes may spoil ovules that become unavailable for cross-fertilization (Bertin and Sullivan 1988; Waser and Price 1991; Santos-Gally *et al.* 2013a; Simón-Porcar *et al.* 2015a). Physical interactions may involve obstruction by an excess of pollen grain deposition on the stigma or pollen tubes growing in the style, from either heterospecific or conspecific pollen (stigma or style clogging; Van der Pijl 1978; Holland and Chamberlain 2007), the reduction of pollen dispersal because of close proximity between stigma and anthers (Lloyd and Yates 1982; Kohn and Barrett 1992; Fetscher 2001) or reduced availability of pollen for outcrossing (Harder and Wilson 1998). However, herkogamy may negatively affect the male and female functions, as anthers and stigmas at different levels increase the likelihood of imprecise pollen transfer between flowers (Darwin 1877; Barrett and Glover 1985; Webb and Lloyd 1986). Dichogamy, on the other hand, may have a disadvantage when stigma receptivity does not overlap temporally with pollen presentation among flowers (Lloyd and Webb 1986).

One mechanism with accurate pollen transfer between the sexual organs of different flowers, which simultaneously promotes outcrossing and avoids self-interference, occurs in heterostylous plants and other reciprocal polymorphisms (Barrett 2002). Heterostyly is characterized by the presence of two (distyly) or three (tristyly) flower morphs with reciprocal positioning in the height of the stigma and the anthers among flowers of different plants within a population (reciprocal herkogamy). Darwin (1877) proposed that the ultimate function of heterostyly is to promote pollination between anthers and stigmas of different morphs, that is cross-fertilization. Lloyd and Webb (1992a, b) extended Darwin’s original hypothesis, proposing an evolutionary model of heterostyly based on phenotypic selection, in which non-reciprocal stylar dimorphism (morphs differ in stigma height but not anther height) was proposed as the intermediate step in the evolution of distyly. Although stylar dimorphism is less frequent than distyly, it occurs in several genera: *Lithodora*, *Glandora*, *Anchusa* (Boraginaceae), *Nivenia* (Iridaceae), *Linum* (Linaceae; Thompson 2005; Ferrero *et al.* 2009; Sánchez *et al.* 2010; Ruiz-Martín *et al.* 2018) and particularly *Narcissus*, in which the style polymorphism is present in at least 12 species (Barrett *et al.* 1996; Baker *et al.* 2000; Barrett and Harder 2005; Pérez-Barrales *et al.* 2006; Santos-Gally *et al.* 2013a).

**Figure 1.**
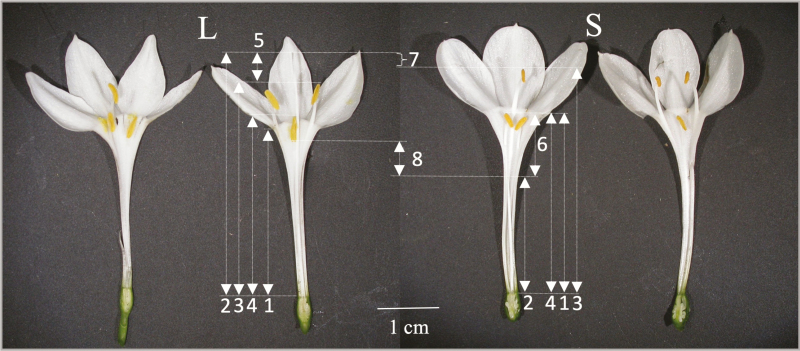
Long- (L) and short- (S) styled morphs of *Narcissus broussonetii*. 1: Floral tube length; 2: stigma length; 3: upper anthers height; 4: lower anthers height; 5: herkogamy of the L-morph; 6: herkogamy of the S-morph; 7: reciprocity of the L-morph; 8: reciprocity of the S-morph.

Stylar dimorphic species exhibit two stylar morphs, long- (L) and short- (S) styled, where the stamens are in a similar position and differ mainly in the height of the stigma. L-morph plants present the stigma above (approach herkogamy) or, as in *Narcissus*, at the same height as the anthers, whereas S-morph plants exhibit the stigma well below the anthers (reverse herkogamy) (Fig. 1). Because there is no reciprocal placement of the stigma and the anthers between morphs and there is a high potential for sexual interference in L-morph flowers given the lack of anther–stigma separation in *Narcissus* (Barrett *et al.* 2000; Arroyo *et al.* 2002), the presence of some other floral traits that prevent sexual interference within the L-morph is expected (Baker *et al.* 2000; Pérez *et al.* 2004; Ferrero *et al.* 2009; Santos-Gally *et al.* 2015). Indeed, recent works have shown that L-morph flowers, but not S-morph flowers, of some stylar dimorphic *Narcissus* species present dichogamy as a mechanism avoiding self-pollination (Cesaro *et al.* 2004; Simón-Porcar *et al.* 2015a). These species have incomplete dichogamy and show some overlap between the presentation of pollen and stigma receptivity, only in the L-morph (Cesaro *et al.* 2004; Simón-Porcar *et al.* 2015a). The stigma receptivity in most protandrous species is initially delayed by the style growth (Lloyd and Webb 1986; Barrett *et al.* 1996). However, dynamics of style growth in protandrous style dimorphic species and the influence on reciprocity, and therefore on self-interference, have not been reported.

*Narcissus broussonetii* presents the weakest reciprocity between sex organs of morphs of any *Narcissus* species, resulting in fewer opportunities for inter-morph crossings, especially between anthers of the L-morph and stigma of the S-morph, because the distance that separates them is almost twice the distance between the anthers of the S-morph and the stigma of the L-morph or the distance between the anthers and the stigma of the L-morph (Santos-Gally *et al.* 2015) (Fig. 1). The ultimate consequence of this arrangement could explain the high frequency of L-morph plants, as found in five of six sampled populations where an excess of L-morph plants was reported (Santos-Gally *et al.* 2015). However, the previous study also showed that the seed set of L-morph plants tended to be lower than that of S-morph plants (Santos-Gally *et al.* 2015), which seems contradictory with the abundance of this morph in natural populations. Because the stigma of L-morph is positioned well above their anthers, L-morph might present dichogamy, as other style dimorphic *Narcissus* species, and hand-pollination on stigmas at different developmental stages might explain the previously observed differences in seed set in inter-morph crosses.

In this study we investigated the occurrence of dichogamy and herkogamy in the style dimorphic *N. broussonetii* and their effect on self-interference avoidance and reciprocity between morphs. First, we estimated the relative growth of sex organs after anthesis and its effect on maturation, and spatial separation and reciprocity between both morphs. Second, we compared the female function of morphs, in terms of the timing of stigma receptivity and effects of flower age on seed production, by carrying out controlled hand-pollinations. Anther dehiscence occurs at flower opening and pollen is exposed to pollinators during the whole flower lifespan, as in other *Narcissus* species; therefore, we did not evaluate experimentally the male function, which is mostly affected by extrinsic factors (Cesaro *et al.* 2004; Medrano *et al.* 2005; Simón-Porcar *et al.* 2015a). Additionally, given the weak reciprocity between the sex organs of morphs of this species, we estimated the genetic diversity parameters of a natural population of *N. broussonetii* to explore their possible differences between morphs as a result of different rates of inbreeding in each morph within the population, for example if morphs are spatially and genetically clumped due to low dispersal, leading to mates occurring mostly within morphs (assortative mating; Hodgins and Barrett 2006; Stehlik *et al.* 2006) or when short-tongued pollinators, which mostly pollinate L-morph flowers, are abundant, favouring assortative mating among L-morph plants (Pérez-Barrales and Arroyo 2010; Santos-Gally *et al.* 2013b, 2015; Simón-Porcar et al. 2014).

## Methods

### Study species and population sampling

*Narcissus broussonetii* is a geophyte endemic of Atlantic central and southern coasts of Morocco (Fernandes 1940), which represents the southernmost range of daffodils. Flowering plants produce up to 15 flowers, which are arranged in an umbel-like inflorescence, from October to early December albeit in a very irregular fashion, in timing and intensity, due to unpredictability of autumn rainfall in this arid sub-Mediterranean region. *Narcissus broussonetii* is a stylar dimorphic species with an unusual combination of perianth traits among daffodils. It has the longest floral tube of all dimorphic species in the genus (26.9 ± 0.5 mm), where the stamens are adnate in two whorls (three stamens in each one) and a virtual lack of a corona (Fig. 1). Most of the identified populations are anisoplethic where the L-morph predominates (56.1–90.8 %; Santos-Gally *et al.* 2013a, 2015). It is self-incompatible but morph-compatible, that is crosses between plants of the same morph render similar seed set than crosses between different morphs (Santos-Gally *et al.* 2015).

Plants used in this study were collected from a natural population in south-western Morocco (29°39′N; 9°26′W, SEV284809) and were transferred to a glasshouse at University of Seville in Spain. We collected plants separated by 1 m or more to avoid repetition of the same genet, as there is some vegetative reproduction. Plants were randomly collected in the population; thus, morph proportion in our sample reflects that in the population and its genetic diversity. We preferred to collect plants from a single population, the largest among those visited, rather than to have a sample collected across smaller populations, to minimize confounding effects of possible among-population differences. Plants were grown in free-draining black plastic pots, containing a standard commercial potting mix (Blumenerde, Gramoflor). Pots were watered until field capacity daily, as it usually occurs in the field during autumnal growing season prior to flowering.

### Relative growth of sex organs, herkogamy and reciprocity between sex organs of morphs

We compared dynamics of style growth between morphs using morphometric measurements of flowers from anthesis until Day 6 (day) to determine the differences in the development of sex organs. In this study anthesis is defined as the time when the perianth opens and sexual organs are presented to floral visitors. Floral longevity of *Narcissus* species ranges from 5 to 20 days (Arroyo and Dafni 1995; Barrett *et al.* 1996; Worley *et al.* 2000; Medrano *et al.* 2005; Simón-Porcar *et al.* 2015a). Flowers were measured until Day 6, because the withering of sex organs and floral tube were visually evident in that day for *N. broussonetii*. We selected the first flower of the inflorescence in all plants because the resources allocated to flowers may vary according to the opening sequence and its position within the inflorescence. Flowers were attached to a flat support with a millimetre scale and photographed (once a day) using a digital camera from the day the flower opened until the sixth day after flower opening. The camera was placed on a tripod perpendicular to the flower so that all flowers were photographed from the same angle. In the S-morph flowers a small and narrow incision on the top of the floral tube was made in order to photograph the stigma. A total of 135 floral images were taken of which 99 correspond to 18 L-morph plants and 36 to 6 S-morph plants. In both cases the number of photos is the same for each day. We measured four traits: floral tube length, style length, upper- and lower-whorl anther height (Santos-Gally *et al.* 2015) (Fig. 1). We measured only one stamen per whorl. The floral tube was measured as a control for potential allometric relations with the sex organs. Style, upper and lower anther lengths were adjusted using the formula described by Baker *et al.* (2000), in which: adjusted data = organ position − *b* (floral tube length − floral tube mean length), where *b* indicates the slope of the regression of organ position and floral tube length. All measurements were taken from digital images of the side view of flowers using ImageJ (Schneider et al. 2012). For each plant we calculated the total growth of floral tube, style, upper and lower anthers as the difference between the length of each trait when the flower opened and their maximum length after the flower opening and the relative growth for each day. We used generalized estimated equations (function GEE in SPSS 2016, version 24; IBM Corp 2016) to account for allometric effects and morph on sex organ (style, upper anthers or lower anthers) growth, during floral lifespan. Each flower was identified and this information was included in the model as a subject variable. Flower age (1- to 6-day) was defined as a within subject factor, because there were repeated measures of each trait. We included morph as a fixed effect and the floral tube growth as a time-varying covariate. Finally, we selected a gamma distribution with log link and to test model effects type III and Wald chi-square statistic.

Spatial separation (mean ± SE) of sex organs was estimated for 1- to 6-day flowers. The herkogamy was calculated as the difference between the stigma height and upper anthers height for L-morph plants and as the difference between the lower anthers height and stigma height for S-morph plants (Fig. 1). The reciprocity (mean ± SE) of sex organs between morphs during the flower lifespan was also estimated for 1- to 6-day flowers, because the heights of anthers and stigmas differed according to flower age. We estimated the reciprocity for each flower age as the difference (mean ± SE) between the stigma height and all the anther heights during the floral lifespan. In contrast with most of other style dimorphic and distylous species, in *Narcissus* the stamens are placed in two whorls. Thus, the reciprocity for the L-morph was estimated as the separation between its stigma height and the upper anther height of the S-morph, and for the S-morph as the separation between its stigma height and the lower anther height of the L-morph (Fig. 1). This method permits comparison between morphs in reciprocity.

### Stigma receptivity

We compared pollen germination on stigmas of different ages, i.e. 1- to 6-day flowers after anthesis to determine the timing of stigma receptivity (Cesaro *et al.* 2004). Plants were grown under glasshouse conditions and caged with a mesh (pore size 0.1 mm) to avoid any interference by pollinating insects. We randomly assigned each of the six treatments (flower age: 1–6 days) to flowers at different positions in the inflorescence for each plant. Flowers were emasculated before anthesis and pollinated (until stigma surface saturation) with a mix of pollen from 1-day flowers of several L- and S-morph plants. We collected flowers and cut the stigmas 1 h after pollination (Cesaro *et al.* 2004). We made a slide preparation for each stigma by squashing and heating it under a cube of fuchsine-stained jelly (Dafni 1992). A total of 107 stigmas of 30 L-morph plants and 62 stigmas of 13 S-morph plants were examined under light microscopy. Finally, we counted the number of germinated pollen grains on the stigma surface.

The effect of flower age on stigma receptivity during floral lifespan was analysed using a generalized linear model (function GEE in SPSS 2016, version 24; IBM Corp 2016). The distribution of the dependent variable (number of germinated pollen grains) was Poisson with log link. We included the morph (L or S) and flower age (1- to 6-day flowers) and their interaction as categorical predictors. To test model effects, we selected type III and Wald chi-square statistic.

### Effect of flower age on seed production

We applied six hand-pollination treatments with flowers of different ages (i.e. 1- to 6-day) to determine the effect of flower age on seed production. The hand-pollination treatment was the same as applied above. The position of each recipient flower on the inflorescence of each treatment was randomly selected and the number of replicates per treatment was balanced across flower positions. Plants were grown outside of the glasshouse and each scape and its inflorescence were caged with a mesh (pore size 0.1 mm). Fruits were harvested 6–8 weeks after hand-pollinations and the number of plump seeds, aborted seeds and undeveloped ovules were counted. A total of 50 L- (260 flowers) and 17 S-morph plants (101 flowers) were used in the experiment, making the number of flowers of each treatment similar for each morph. Because the total number of ovules in the ovary is not the same between flowers in different positions in the inflorescence, we estimated the seed set using the formula: seed set = number of seeds developed/total number of ovules in the ovary.

The effect of flower age on seed production was analysed using generalized estimated equations GENLIN for proportion data (function GEE in SPSS 2016, version 24; IBM Corp 2016). A binomial error distribution was used for the dependent variable (number of ovules converted into seeds) and flower age (days), morph and their interaction as categorical predictors. Repeated measurements (plants) and the position of the flower within an inflorescence, which may affect resources available for seed development, were included in the analyses as subject and within-subject variables, respectively. To test model effects, we specified type III sum of squares, chi-square statistic and kernel as a log quasi-likelihood function.

### Population genetic diversity parameters

We used 10 microsatellite loci to determine the heterozygosity, inbreeding coefficients and other genetic diversity parameters within the population and between morphs to test for possible genetic differences between morphs. These microsatellite loci were developed for *N. papyraceus* and have been tested in other *Narcissus* species (Simón *et al.* 2010; Barranco *et al.* 2019). We collected two fresh leaves from each of the plants maintained in the glasshouse (a total of 74 L- and 17 S-plants). Genomic DNA was extracted from silica-dried leaves using the DNeasy Plant Mini Kit (Qiagen Inc., Chatsworth, CA, USA) and quality of DNA was estimated with a Nanodrop 2000 spectrophotometer (Desjardins and Conklin 2010). Polymerase chain reaction was performed in 25 µL of reaction mixture containing 50 ng µL^−1^ DNA, 1× PCR buffer, 1.5 mM M_g_Cl_2_, 0.1 µM forward primer, 0.1 µM reverse primer, 0.2 mM mix dNTPs and 1.25 U Tap polymerase. Samples were incubated in a thermal cycler TC-5000. Thermocycling conditions for the microsatellite loci were 5 min at 94 °C, followed by 45 cycles at 94 °C for 30 s, 50 °C for 30 s (annealing) and 72 °C for 30 s (extension) with a final extension at 72 °C for 5 min. To determine whether amplification was successful we ran 1 µL of the product in an agarose gel. Sequencing was performed using Applied Biosystems Prism model 3700 automated sequencer.

### Genetic data analysis

Results were analysed using Peak Scanner v1.0 software and LIZ 500 size standard. Genotyping errors, such as short allele dominance (large allele dropout), stutter peaks and typographic errors were detected with Micro-Checker v 2.2.3 (Van Oosterhout *et al.* 2004), using 10 000 permutations and 95 % confidence interval. For the non-amplified alleles (null alleles), the frequencies were calculated with Null Allele Estimator (for non-equilibrium populations; Van Oosterhout *et al.* 2006) using the inbreeding coefficient (average for all microsatellite loci) and homozygote frequency observed.

Average number of alleles observed per locus (*A*) and allelic richness (mean number alleles per locus corrected for the minimum sample size; *R*_s_) were estimated using FSTAT version 2.3.9 (Goudet 1995). The *P*-values for the Hardy–Weinberg equilibrium (HWE), inbreeding coefficients *F*_ST_ (mean inbreeding coefficient of subpopulations of L- and S-morph plants, respectively, relative to the total population) and *F*_IS_ (mean inbreeding coefficient of L- and S-morph individuals, respectively, relative to the subpopulations of L- and S-morph plants), number of private alleles observed per locus (*PA*), observed heterozygosity (*H*_o_) and gene diversity (*H*_e_) were estimated with GenAlEX v. 6.501 software (Peakall and Smouse 2012). We conducted an ANOVA (in SPSS 2016 version 24) to compare the genetic *A*, *R*_s_, *H*_o_ and *H*_e_, between morphs, using 10 000 bootstrap replicates and 95 % confidence interval. The genetic diversity parameters and inbreeding coefficients were estimated for L- and S-morph plants separately, to compare between them. The genetic structure of L- and S-morph plants of *N. broussonetii* was explored using Bayesian analyses with STRUCTURE v2.3.4 (Pritchard *et al.* 2000; Hubisz *et al.* 2009). This software assigns individuals (or populations) to clusters (*K*) using genotype data (unlinked markers). In our analyses, we specified two different clusters (*K* = 2), to test whether L- and S-morph plants can be classified into two independent clusters according to their genetic data. We ran the analyses under correlated allele frequencies and admixture models. We specified a burn-in period of 50 000 interactions and 20 000 Markov Chain Monte Carlo (MCMC) repetitions after the burn-in. Finally, we repeated the simulation 10 times for a number of clusters *K* = 2. Individual’s membership fraction was estimated for all the plants included in the analyses (74 L and 17 S).

## Results

### Relative growth of sex organs, herkogamy and reciprocity between sex organs of morphs

**Figure 2.**
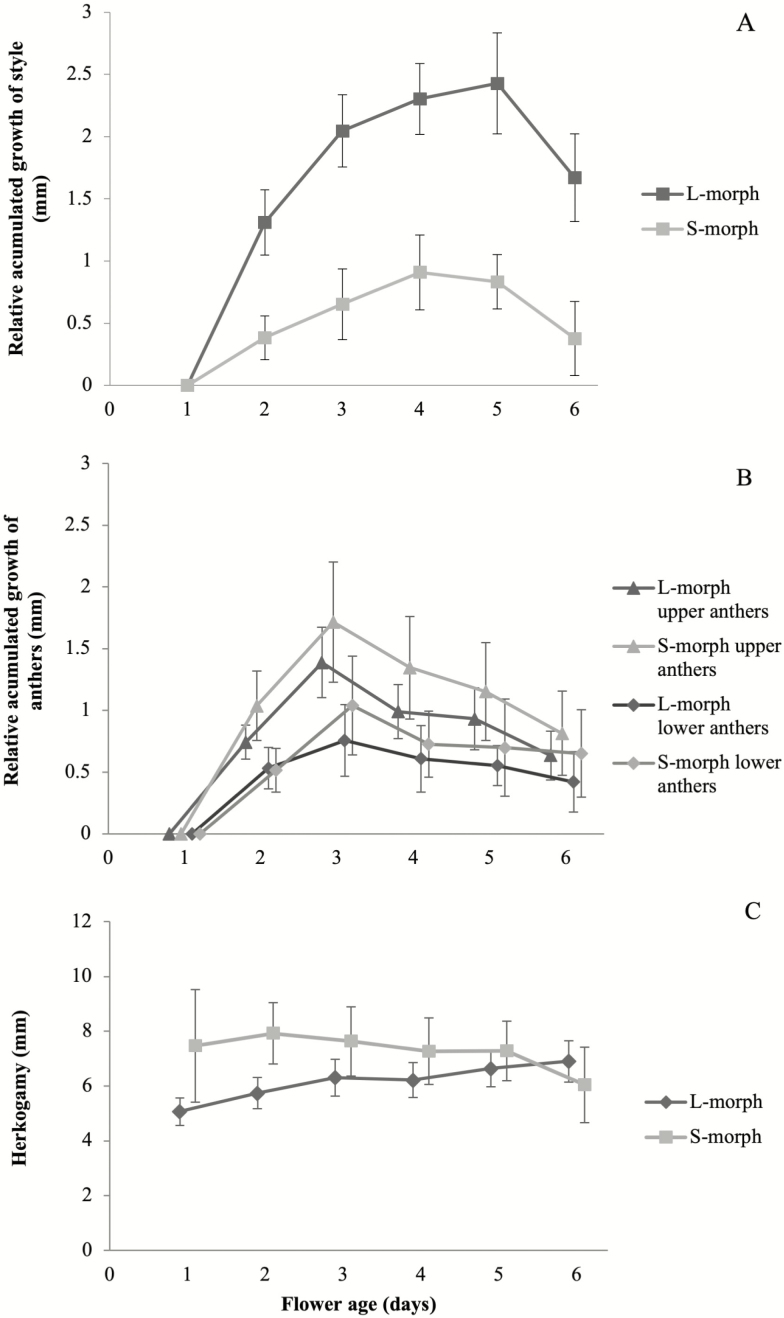
Relative accumulated growth (mean ± SE) of sex organs after the anthesis in 1- to 6-day flowers of *Narcissus broussonetii*. (A) Relative accumulated growth of style. (B) Relative accumulated growth of upper and lower anthers. (C) Herkogamy (mean ± SE) of stigma and upper anther heights for the L-morph and of lower anthers and stigma heights for the S-morph.

Upper and lower anther growth was not correlated with floral tube growth nor did it differ between morphs (Table 1). However, style length was obviously different between morphs, independent of floral tube growth (Table 1). The floral tube grew a total of 1.1 ± 0.2 mm and 0.8 ± 0.3, respectively, for the L- and S-morph, from flower opening until the fourth day after anthesis **[see**Supporting Information—Fig. S1**]**. Upper anthers grew a total of 0.9 ± 0.3 mm for the L-morph plants and 0.6 ± 0.5 mm for the S-morph plants and lower anthers grew a total of 1.0 ± 0.4 mm for the L-morph plants and 0.8 ± 0.4 mm for the S-morph plants. Upper and lower anthers grew until the fourth day after anthesis (Fig. 2B). Styles grew a total of 2.4 ± 0.3 mm for the L-morph plants and 0.7 ± 0.5 mm for the S-morph plants. Three days after the flower opened the style grew a total of 1.9 ± 0.3 mm for the L-morph plants and 0.4 ± 0.5 mm for the S-morph plants (Fig. 2A). We also recorded the withering of sex organs after they reached their maximum height (Fig. 2A and B; **see**Supporting Information—Fig. S1).

**Table 1.** Results of the generalized estimated equations analysis for upper and lower anthers and style growth as dependent variable, morph as fixed effect, growth of the floral tube as a covariate and flower age as repeated measures.

		Sexual organs growth after anthesis		
		χ ^2^ Wald	*P*-value	d.f.
Upper anthers	Morph (L and S)	2.4	<0.115	1
	Floral tube	0.8	<0.773	1
Lower anthers	Morph (L and S)	0.1	<0.696	1
	Floral tube	2.4	<0.115	1
Style	Morph (L and S)	6.5	<0.010	1
	Floral tube	0.4	<0.507	1

**Figure 3.**
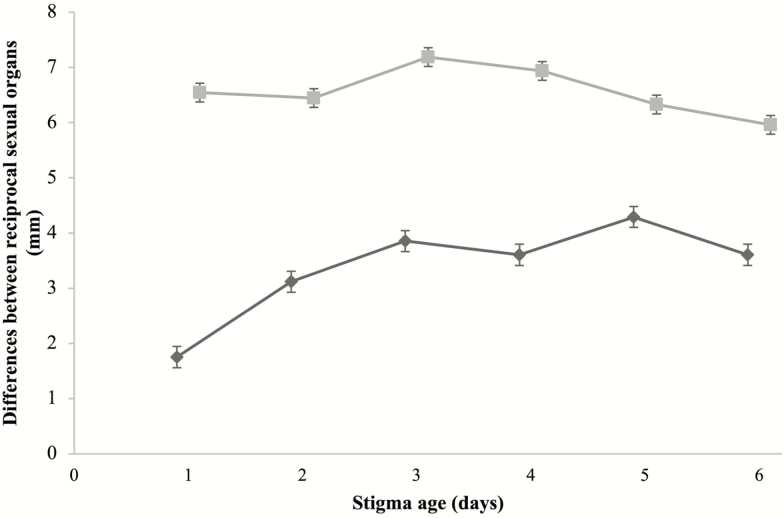
Differences between reciprocal sexual organ length (mean ± SE mm) from 1- to 6-day flowers of *Narcissus broussonetii.* Diamonds represent the differences between the stigma height of the L-morph and upper anthers height of the S-morph and squares the difference between the lower anthers height of the L-morph and stigma height of the S-morph.

The greatest spatial separation between anthers and stigmas was in 2-day flowers for the S-morph (7.9 ± 1.3 mm) and 3-day flowers for the L-morph (6.3 ± 0.7 mm). The lowest spatial separation between anthers and stigmas was in 1-day flowers for the L-morph (5.0 ± 0.5 mm) and 6-day flowers for the S-morph (6.0 ± 1.3 mm). Herkogamy was higher in S- than L-morph flowers for 1- to 5-day flowers (Fig. 2C). Reciprocity was variable across the flower lifespan but L-morph plants always presented stronger reciprocity than S-morph plants. The strongest reciprocity for the L-morph was at flower opening (1.7 ± 0.2 mm) and the weakest was in 3-day flowers (3.8 ± 0.2 mm), when the styles reached their maximum length. The strongest and weakest reciprocity for the S-morph were in 3-day (7.2 ± 0.2 mm) and 6-day flowers (5.9 ± 0.2 mm), respectively (Fig. 3).

### Stigma receptivity

**Figure 4.**
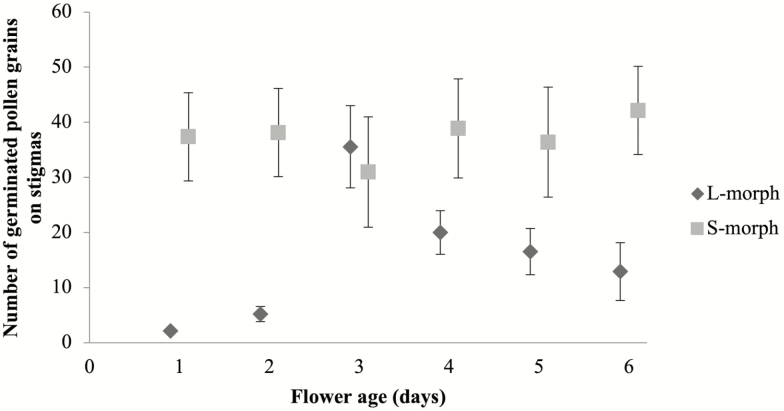
Germinated pollen grain number (mean ± SE) on stigmas of L- and S-morphs of *Narcissus broussonetii* after hand-pollinations on 1- to 6-day flowers. Statistical results are given in Table 2A.

The number of germinated pollen grains differed significantly between morphs, flower age and their interaction (Table 2A). The stigma receptivity was high for S-morph flowers throughout the entire observation period (6 days). The stigma receptivity of L-morph flowers was very low during the first 2 days, peaked in the third day and decreased during the fourth to sixth days (Fig. 4).

**Table 2.** Results of the generalized linear model analysis for stigma receptivity (A) and seed set (B) data as dependent variable, morph flower age and their interaction as fixed effects.

	χ ^2^ Wald	*P*-value	d.f.
A: Stigma receptivity (number of germinated pollen grains)			
Morph (L and S)	851.1	<0.001	1
Flower age (1- to 6-day)	360.0	<0.001	5
Interaction: Morph (L and S) × Flower age (1- to 6-day)	476.7	<0.001	5
B: Seed set			
Morph (L and S)	12.1	0.001	1
Flower age (1- to 6-day)	24.1	<0.001	5
Interaction: Morph (L and S) × Flower age (1- to 6-day)	3.8	0.568	5

### Effect of flower age on seed production

**Figure 5.**
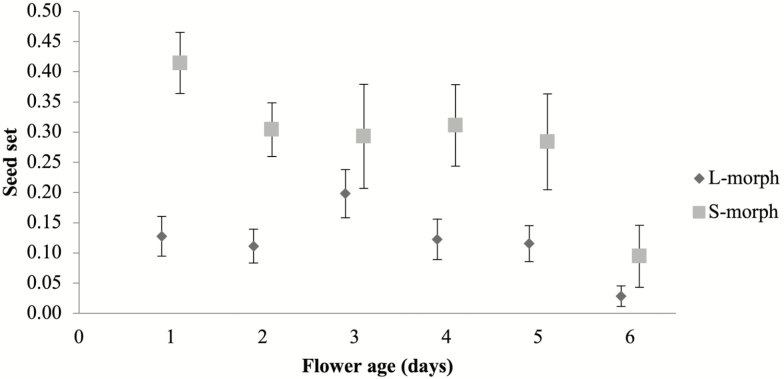
Seed set (mean ± SE) of L- and S-morphs of *Narcissus broussonetii* after hand-pollinations on 1- to 6-day flowers. Statistical results are given in Table 2B.

The number of ovules converted into seeds differed between morphs and flower ages (Table 2B). S-morph plants produced significantly more seeds than L-morph plants. Flowers of both morphs tend to produce most seeds when pollinated during the first day of high stigma receptivity (Day 1 for S-morph flowers, Day 3 for L-morph flowers) and fewest seeds when pollinated in the sixth day of flower lifespan (Fig. 5).

### Population genetic diversity parameters

**Figure 6.**
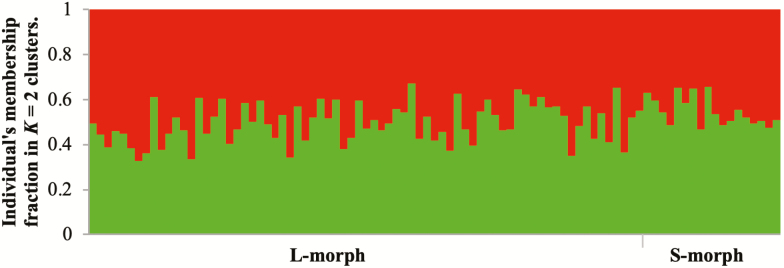
Genetic structure of L- and S-morphs of *Narcissus broussonetii.* Each individual is represented by a vertical line, which is partitioned into two colours that represent the individual’s membership fraction in *K* = 2 clusters.

The Null Allele Estimator analysis showed no presence of null alleles in any locus. The population was not in HWE for any locus and two of the 10 microsatellite loci were monomorphic, A131 and B104 **[see**Supporting Information—Table S1**]**. The L- and S-morph plants only showed significant differences between morphs in the average number of alleles and private alleles per locus (Table 3). Allelic richness (*R*_s_), *H*_o_ and *H*_e_ did not differ between morphs (Table 3). Inbreeding coefficients, *F*_ST_ and *F*_IS_, were similar between morphs and close to 0 (Table 3). The L- and S-morph plants were not assigned to independent clusters in the STRUCTURE analysis (Fig. 6). The average individual membership fraction was 0.50 ± 0.01 in both clusters for L- and S-morph plants. Posterior probabilities of *K* were consistent across the 10 repetitions, suggesting that the burn-in and run periods were adequate.

**Table 3.** Population genetic parameters (mean ± SD) and inbreeding coefficients (*F*_ST_ and *F*_IS_) of long- (L) and short- (S) morphs in a population of *Narcissus broussonetii*. Sample size (*N*), number of alleles (*A*), number of private alleles (*PA*), allelic richness (*R*_s_), observed heterozygosity (*H*_o_), genetic diversity (*H*_e_) and *P*-values of the ANOVA between morphs for all parameters. *Significant differences between morphs (*P* < 0.05).

Morph	*N*	*A*	*PA*	*R*_s_	*H*_o_	*H*_e_	*F*_ST_	*F*_IS_
L	74	5.9 ± 1.0*	2.7 ± 0.7*	4.2 ± 0.6	0.61 ± 0.11	0.53 ± 0.09	0.05 ± 0.00	–0.16 ± 0.00
S	17	3.2 ± 0.4	0	4.2 ± 0.6	0.58 ± 0.10	0.47 ± 0.08	0.05 ± 0.00	−0.22 ± 0.00

## Discussion

### Functional implications of herkogamy and dichogamy in *Narcissus broussonetii*

Our study highlights the importance of considering herkogamy and dichogamy together when studying sex conflicts within the flower of a self-incompatible and morph-compatible style dimorphic species, and their effects on the reciprocity between morphs and reproductive success. *Narcissus broussonetii*, the daffodil with the southernmost range, presents the highest average herkogamy of all style dimorphic species in the genus (Arroyo and Dafni 1995; Baker *et al.* 2000; Arroyo *et al.* 2002; Pérez-Barrales *et al.* 2006; Santos-Gally *et al.* 2015). The herkogamy is higher in S- than L-morph flowers (Fig. 2C), probably because approach herkogamy is more efficient at avoiding self-interference than reverse herkogamy, a rare condition in flowering plants (Webb and Lloyd 1986). Moreover, our results showed a gradual increase in the herkogamy for L-morph flowers during the life of the flower, which is principally caused by the higher growth of the L-style after flower opening (Fig. 2A). Among heterostylous species for which data on style length variation are available, results indicate that styles of L-flowers have longer cells than the styles of S-flowers (Richards and Barrett 1992; Cohen 2016). However, cell size is not sufficient to account for style length variation and thus cell number, and its proliferation must also differ. Indeed, in distylous Rubiaceae two alternatives in the development of the style have been described: a differential relative growth rate or a differential time of growth inhibition (Richards and Barrett 1992; Faivre 2000). According to our results, *N. broussonetii* exhibits differential relative growth rates of the styles between morphs after flower opening, as styles begin to grow at the same ages for S- and L-morph flowers (Fig. 2A). However, more studies focused in floral ontogeny of style dimorphic *Narcissus* species will be essential to clarify whether the style dimorphism in this genus is a consequence of a different growth rate of the style. The higher growth rate of the style in L- versus S-flowers after anthesis might be the cause of the delay in the stigma receptivity observed in L-flowers, as it has been described for other protandrous species (Lloyd and Webb 1986; Snow and Grove 1995; Barrett *et al.* 1996; Langenberger and Davis 2002). The stigmas of L-flowers of *N. broussonetii* presented a delay in their receptivity of up to 3 days (Fig. 4). However, the stigmas of the S-flowers were receptive at anthesis. The differences in stigma receptivity between morphs were consistent with the patterns of seed production according to each flower age evaluated (Fig. 5). The seed set was similar for both morphs only in 3-day flowers, when the style of L-flowers reached its maximum height, and there were not differences in stigma receptivity between morphs. This observation might explain why previous reports have shown that the seed set of L-flowers tended to be lower than that of S-flowers (Santos-Gally *et al.* 2015). Finally, the upper anthers dehisced just after anthesis in both morphs, as has been reported for other *Narcissus* species (Barrett *et al.* 1996; Cesaro *et al.* 2004; Medrano *et al*. 2012; Simón-Porcar *et al.* 2015a). This is not the only style dimorphic species in the genus in which herkogamy and dichogamy are present. *Narcissus papyraceus*, in the same clade (Santos-Gally *et al.* 2012), and *N. assoanus* showed a delay in the stigma receptivity only in L-flowers and some herkogamy, particularly in the S-flowers (Cesaro *et al.* 2004; Simón-Porcar *et al.* 2015a). However, *N. serotinus*, which is the closest relative of *N. broussonetii* (Santos-Gally *et al.* 2013a), exhibits style monomorphism, where the upper anthers and the stigma are close to each other (0.91–1.1 mm; Díaz-Lifante and Andrés-Camacho 2007) but there is no information regarding stigmatic receptivity. Therefore, the occurrence or absence of high herkogamy and dichogamy in *Narcissus* species might represent a joint strategy along the phylogeny of this genus, as has been described for other floral traits (Santos-Gally *et al.* 2013a), which might be related with a shift to selfing in monomorphic species or with sex interference avoidance in dimorphic species.

Herkogamy and dichogamy occur combined with self-incompatibility in *N. broussonetii*. These mechanisms play an important role in helping to avoid the negative consequences of self-interference. This is especially important in species that have late ovarian self-incompatibility with ovule degeneration, as in most self-incompatible *Narcissus* species (Dulberger 1964; Barrett *et al.* 1997; Sage *et al.* 1999; Arroyo *et al.* 2002; Santos-Gally *et al.* 2015; Simón-Porcar *et al.* 2015a). However, the high herkogamy of *N. broussonetii* determines that this species presents the weakest reciprocity between the sexual organs of morphs of all *Narcissus* species (Santos-Gally *et al.* 2015). S-morph plants exhibit weaker reciprocity than L-morph plants, and the reciprocity of this morph is constant across the flower lifespan (Fig. 3). However, the styles of L-morph plants are very close in height to the upper anthers of S-morph plants at the flower opening and after 3 days when the style of L-morph plants reaches it maximum size and is receptive, reciprocity decrease because difference between stigma height of the L-morph plants and upper anther height of the S-morph plants is twice (Fig. 3).

This study showed how S- and L-morph plants of *N. broussonetii* present different mechanisms to avoid sexual interference. L-morph plants exhibit a steady increment in herkogamy, which produces a delay in the stigma receptivity to avoid sexual interference during the first 2 days after anthesis. This increase in herkogamy is mainly produced by a higher growth of the style, which has negative consequences on the reciprocity between the stigmas and upper anthers of L- and S-morph plants, respectively. S-morph plants present higher herkogamy between their lower anthers and stigma heights, probably to avoid sexual interference within the flowers of this morph. The higher herkogamy of the S-morph also has negative consequences in the reciprocity between its stigma and the lower anthers of the L-morph. In any case, in style dimorphic *Narcissus* species, avoiding self-interference between sexual functions, seems to be more critical for successful outcrossing than improving morph reciprocity. However, self-interference might be stronger in L-morph flowers, as the stigma is initially closer to the upper stamen whorl and self-pollination might result in high ovule discounting, due to late-acting self-incompatibility (Cesaro *et al.* 2004). The stigmas of S-morph flowers never pass through the anther levels in the flower; thus, self-interference might be reduced. In contrast, lack of reciprocity does not seem to be a strong limitation, given that even *Narcissus* species with limited reciprocity show high rates of disassortative mating, despite the fact that there is within-morph compatibility (Simón-Porcar *et al.* 2015b).

### Genetic diversity of morphs

The microsatellite loci developed for *N. papyraceus* and used in several other stylar dimorphic species of this genus (Simón *et al.* 2010; Simón-Porcar *et al.* 2015c; Barranco *et al.* 2019) have been useful estimating the heterozygosity, inbreeding coefficients and other genetic population parameters between morphs in a natural population of *N. broussonetii*. The estimated genetic diversity parameters for all the plants included in the study (Table 3) were lower than those obtained for *N. papyraceus* (Simón-Porcar *et al.* 2015c). These differences between species are probably due to the much lower population size and smaller geographic range of *N. broussonetii* compared to widespread *N. papyraceus* (Santos-Gally *et al.* 2015; Simón-Porcar *et al.* 2015c), but further work in more populations and species is needed to confirm the influence of these factors across species.

Our results did not show genetic differences between morphs in the population (Table 3). Morphs only differ in the number of alleles per locus and number of private alleles, but when we corrected these parameters for the sample size of each morph (estimating the allelic richness) there were no longer any differences (Table 3). Heterozygosity was similar for both morphs, and inbreeding coefficients (*F*_ST_ and *F*_IS_) were close to 0, suggesting that the population is not structured and mating between plants of different morphs occurs widely. In consequence, L- and S-morph plants were not assigned to different clusters depending on their genetic data. We observed that all plants were assigned to clusters (*K* = 2*m*) with similar values of individual membership fraction (Fig. 6). These results also suggest that the population is not genetically structured by the morphs and the alleles and their frequencies are similar between both morphs. The population was not in HWE for any locus supporting a prevalence of assortative or disassortative mating instead of random mating. This result has been observed for other *Narcissus* species (Simón *et al.* 2010; Simón-Porcar *et al.* 2015b; Barranco *et al.* 2019). More studies involving maternal and paternal origins of seeds in natural pollination conditions will be necessary to clarify which type of mating (assortative or disassortative) is predominant in wild populations of *N. broussonetii* and the effect in the maintenance of style dimorphism (Simón-Porcar *et al.* 2015b).

In field conditions, the fecundity of both morphs has not been determined. It would be interesting to test if the delay in the stigma receptivity of L-morph plants produces negative consequences in the fecundity of this morph in the field; because the flower is receptive during a shorter period of time (Fig. 4). It is surprising the prevalence of this morph in all sampled populations from Santos-Gally *et al.* (2015) given this result (Figs 4 and 5). It seems to indicate that L-morph plants of *N. broussonetii* might transmit more of their genes to the progeny through their pollen than their ovules. This gender specialization could be explored with paternity analysis using microsatellite markers (already available) and it will be the first case of gender specialization observed in *Narcissus* species, where the prevalence of disassortative mating and no gender differentiation is the norm (Hodgins and Barrett 2006; Simón-Porcar *et al.* 2015b).

In conclusion, this study demonstrates that anther–stigma reciprocity, a kind of herkogamy involving two sex morphs in a population, is strongly affected by dichogamy. Both mechanisms are thought to decrease sexual interference in flowers, with reciprocal herkogamy suggested as the strongest in promoting the establishment of style dimorphism, aided by the role of active pollinators promoting disassortative effective pollination (Lloyd and Webb 1992b; Simón-Porcar *et al.* 2015b). Here we propose that dichogamy might alter the outcomes of this mechanism, and might explain in part, together morph-compatibility, the excess of L-morph plants in many style dimorphic species in *Narcissus*.

## Supporting Information

The following additional information is available in the online version of this article—

**Table S1.** Primer screening in a population of *Narcissus broussonetii*. Sample size (*N*), number of alleles (*A*), allelic richness (*R*), observed heterozygosity (*H*_o_), genetic diversity (*H*_e_), inbreeding coefficient (*F*) and *P*-values for the Hardy–Weinberg equilibrium test are given for each marker.

**Figure S1.** Relative accumulated growth (mean ± SE) from 1- to 6-day flowers of floral tube of *Narcissus broussonetii*.

plz038_suppl_Supplementary_Material

## Sources of Funding

This work was supported by Ministerio de Economía y Competitividad de España (CGL2009-12565 and CGL2013-45037 PGC2018-099608-B-I00) and Plan Andaluz de Investigación (RNM-210). D.B. was supported by fellowships from Ministerio de Economía y Competitividad de España (BES-2014-067795 and EEBB-I-16-10587).

## Contributions by the Authors

J.A. and R.S.-G. conceived and designed the experiments. D.B. and R.S.-G. performed the experiments. D.B. analysed the data. All authors contributed to discussions, writing and approved the manuscript.

## Conflict of Interest

None declared.
