# Supplementary material for: Avoiding sexual interference: herkogamy and dichogamy in style dimorphic flowers of Narcissus broussonetii (Amaryllidaceae)
Source: AoB Plants. 2019 Aug 9;11(4):plz038. doi: 10.1093/aobpla/plz038 (PMC6735907; doi:10.1093/aobpla/plz038)
Supplement: plz038_suppl_Supplementary_Material [file plz038_suppl_supplementary_material.pdf]

Table S1. Primer screening in a population of *Narcissus broussonetii*. Sample size (*N*), number of alleles (*A*), allelic richness (*Rs*) observed heterozigosity (H_0), genetic diversity (H_e), inbreeding coefficient (*F*) and *P*-values for the Hardy-Weinberg equilibrium test are given for each marker.

Locus	*N*	*A*	*Rs*	H_o	H_e	*F*	HWE
A5	91	8	5.74	0.86	0.71	-0.21	0.000*
A109	91	3	2.99	0.81	0.59	-0.37	0.000*
A116	91	10	6.86	0.68	0.65	-0.04	0.014*
A121	90	7	4.86	0.64	0.58	-0.10	0.000*
A131	91	1	1	0.00	0.00	-	-
A134	88	7	5.03	0.89	0.67	-0.35	0.000*
B7	91	9	4.92	0.86	0.60	-0.42	0.000*
B104	91	1	1	0.00	0.00	-	-
B112	91	5	4.29	0.66	0.71	0.08	0.028*
B131	90	8	5.45	0.67	0.73	0.09	0.008*

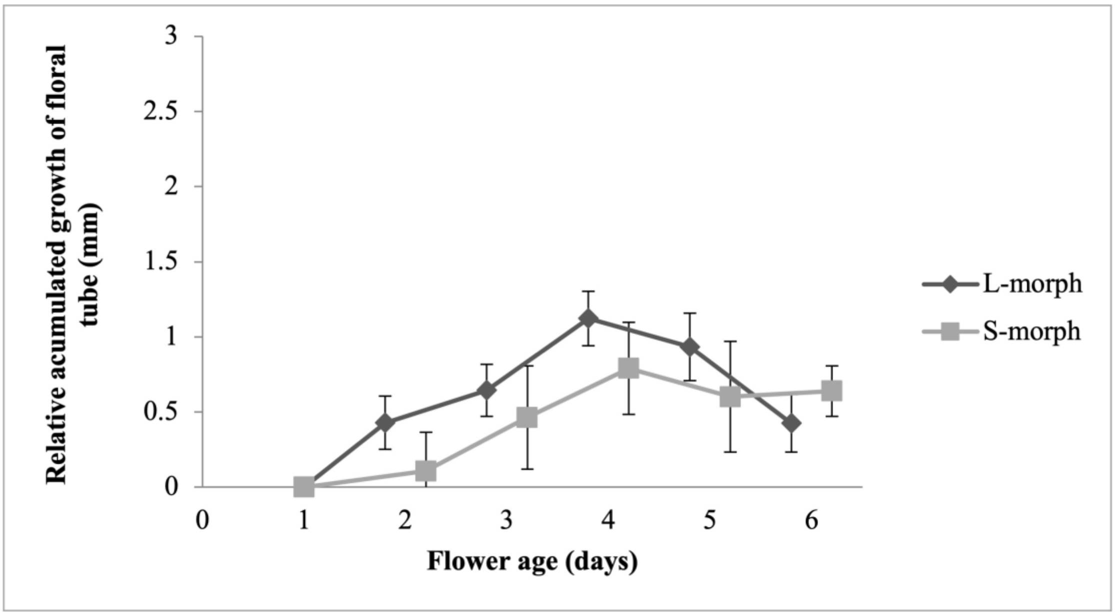

Figure S1. Relative accumulated growth (mean ± SE) from 1- to 6-d flowers of floral tube of *Narcissus broussonetii*.
